# Successful *en bloc* endoscopic full-thickness resection of a giant cervical esophageal leiomyoma originating from muscularis propria

**DOI:** 10.1186/s13019-019-0847-5

**Published:** 2019-01-21

**Authors:** Sumin Zhu, Jie Lin, Shu Huang

**Affiliations:** 1 Department of Gastroenterology, The Second Affiliated Hospital, Nanjing Medical University, Nanjing, China; 2 Department of Gastroenterology, People’s Hospital of Lianshui, 6 Hongri Road, Huaian, China

**Keywords:** Endoscopic full-thickness resection, Esophageal leiomyoma, Purse-string suture, Single-channel endoscopy

## Abstract

**Background:**

Esophageal leiomyomas, the most common benign primary tumors of the esophagus, are esophageal subepithelial lesions treated by surgery traditionally. In recent years, endoscopic submucosal dissection and related endoscopic treatment techniques are adopted by endoscopists to resect gastrointestinal submucosal tumors. But if a giant esophageal leiomyoma approaches the esophagus entrance and originates from the deep layer of muscularis propria, it will be difficult for both endoscopic resection and surgical treatment. Especially, endoscopic resection has a high risk of huge perforation difficult to be sutured.

**Case presentation:**

A 72-year-old man with dysphagia underwent gastroscopy examination which indicated a large submucous eminence lesion, about 18–24 cm from the incisors. Endoscopic ultrasonography revealed the lesion was hypoechoic and originated from the muscularis propria with a clear boundary. The patient refused invasive surgical resection. Then, an *en bloc* endoscopic full-thickness resection was performed, which perforation was successfully closed with purse-string sutures using a novel endoloop device through standard single-channel endoscopy. Histopathologic examination showed an esophageal leiomyoma.

**Conclusion:**

This endoscopic procedure may be an alternative to avoid surgery for the removal of a giant upper esophagus tumor from muscularis propria layer.

## Background

Esophageal leiomyoma is the commonest benign primary tumor arising from smooth muscle cells of the esophagus [1], and shows a subepithelial lesion in gastroscopy. The methods of treatment for esophageal leiomyoma include surgical enucleation, esophagectomy, and endoscopic resection [1]. With the development of endoscopic technology, the more endoscopic techniques have been proven to be feasible and micro-traumatic for the resection of esophageal leiomyoma, such as submucosal tunneling with endoscopic removal (STER) and endoscopic full-thickness resection [2]. For the choice of excision methods, the size and location of esophageal leiomyoma are the key factors. If a giant esophageal leiomyoma approaches the esophagus entrance and originates from the deep layer of muscularis propria, it will be difficult for both endoscopic resection and surgical treatment. Especially, endoscopic resection has a high risk of huge perforation difficult to be sutured. Here, we will give a case report of successful *en bloc* endoscopic full-thickness excavation of a giant cervical esophageal leiomyoma originating from muscularis propria.

## Case presentation

**Fig. 1:**
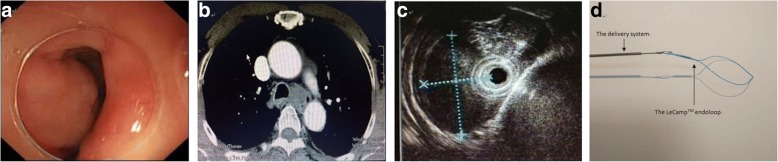
Evaluation of the SMT and the LeCamp™ endoloop device. **a** an endoscopic image revealed a protruding submucous lesion with a smooth surface. **b** CT image showing a 1.8 cm × 5.2 cm × 2.9 cm soft tissue mass. **c** EUS image showing a hypoechoic mass originated from the muscularis propria layer. **d** the LeCamp™ endoloop and its delivery system

A 72-year-old man with dysphagia was admitted to the hospital. Gastroscopy revealed a large submucous eminence lesion, about 18–24 cm from the incisors (Fig. 1a). CT examination indicated a 1.8 cm × 5.2 cm × 2.9 cm soft tissue mass in the upper esophageal wall (Fig. 1b). Endoscopic ultrasonography findings: the lesion was hypoechoic and originated from the muscularis propria with a clear boundary (Fig. 1c). The patient refused invasive surgical resection and the informed consent was obtained for endoscopic submucosal excavation (ESE).

**Fig. 2:**
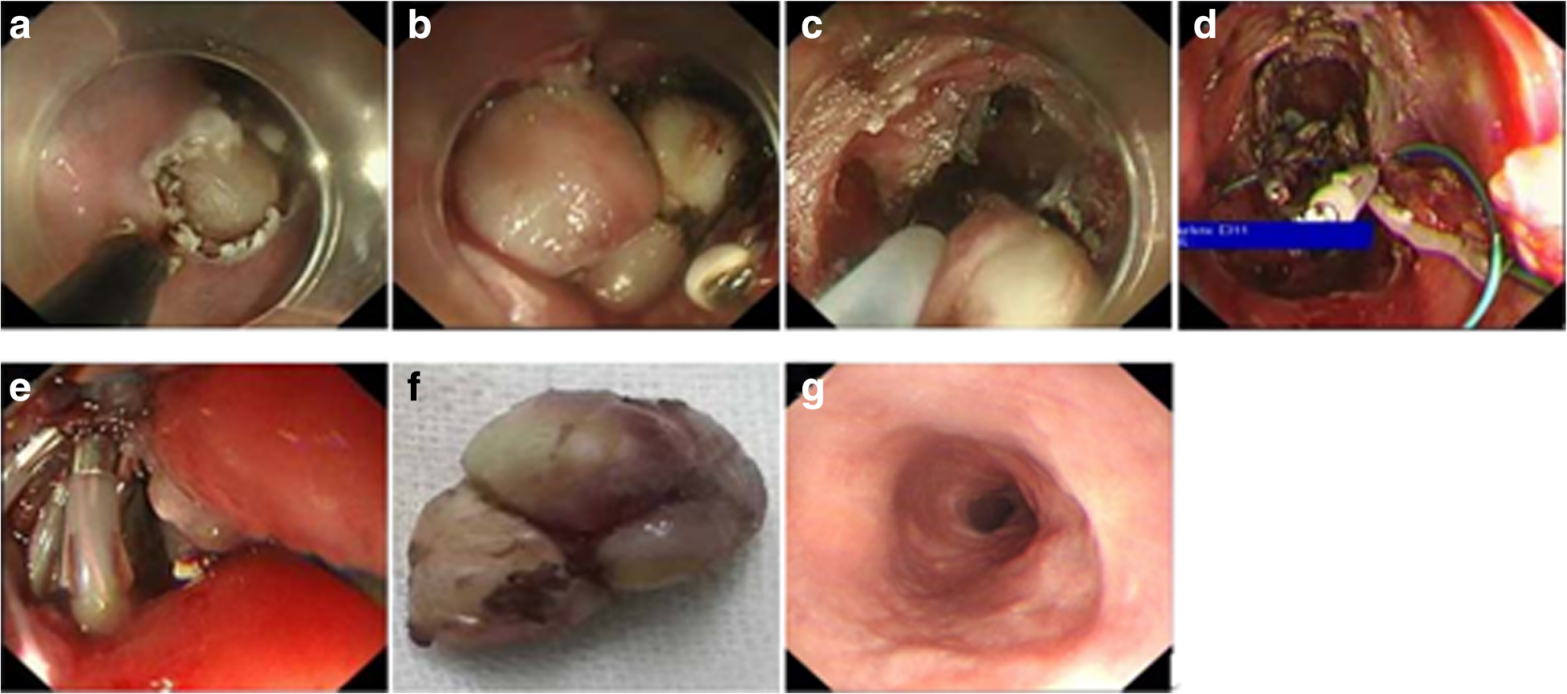
Endoscopic views showing: **a** and **b** the body of the tumor exposed gradually; **c** endoscopic full-thickness resection to achieve *en bloc* resection; **d** the LeCamp™ endoloop being anchored onto the full thickness of the edge of the perforation with the endoclips; **e** successful closure of the perforation following tightening of the nylon string to pull all the clips together; **f** the extracted tumor; **g** no esophageal stricture 2 months later

After marking and submucosal injection, an arc incision along the longitudinal direction of the esophagus was made to avoid the enlargement of the defect. Since the lesion was clinging to the esophageal adventitia, we performed full-thickness resection to achieve *en bloc* resection (Fig. 2a, b, c and f). Taking into account the difficulty of placing the gastrointestinal decompression tube, we placed the guide wire in advance. The perforation was closed with purse-string sutures using a novel LeCamp™ endoloop (Leo Medical Co., Ltd., China) (Fig. 1d), which was inserted into the perforation site through the biopsy channel. After adjusting the location and angle of the endoloop, it was anchored symmetrically onto the full thickness of the perforation’s margin with the clips (Fig. 2d). Then the removable hook was inserted and connected with the endoloop, which was tightened by slight pulling all the edges together. Subsequently the hook was removed from the endoloop and the perforation was closed (Fig. 2e). A 20-gauge needle was used to relieve the subcutaneous emphysema during and after the procedure. Finally, a gastroduodenal decompression tube was placed. Pathological diagnosis of the tumor was leiomyoma. The patient’s postoperative recovery was uneventful. No esophageal stricture was observed 2 months later (Fig. 2g).

## Discussion and conclusions

Although esophageal leiomyoma is rarely symptomatic, the most common symptoms are dysphagia, pain, and weight loss [1]. If esophageal leiomyoma is symptomatic or if there is a strong clinical suspicion for malignancy, it should be resected [1]. The conventional surgical approach especially for giant esophageal leiomyoma has been open thoracotomy or tumor resection through thoracoabdominal incision and sometimes along with gastroesophagostomy [3]. Minimally invasive surgery, VATS (video assisted thoracoscopic surgery), for enucleation of esophageal leiomyoma has been reported since 1992 and it has widely gained acceptance in the last few years [3]. With the development of endoscopic resection technology, STER and ESE are nowadays both adopted by endoscopists to resect gastrointestinal submucosal tumors (SMTs) [2]. STER offers advantages of a very good view of the dissection through the submucosal tunnel, and submucosal tunneling maintains the integrity of the mucosal layer over the tumor [4]. The main features of this case included big size, locating in the upper esophagus (adjacent esophagus entrance) and originating from the deep layer of muscularis propria. Hence, no enough length to construct an esophageal tunnel limited the use of STER in this case. Direct ESE also has a high risk of huge iatrogenic perforation difficult to be sutured with conventional endoclips. Closure could not even be achieved using fully covered self-expandable metal stent because this lesion is too close to the esophagus entrance.

The secure closure has been considered to be the major obstacle of endoscopic iatrogenic perforation and endoscopic full-thickness resection. The over-the-scope clip (OTSC) system or the Overstitch endoscopic suturing device may be selected, but the high cost and difficult operation limits their application. Recently, the endoscopic purse-string suture method via a two-channel endoscopy is proved to be an effective and safe technique for the closure of large perforation [5]. However, the two separate channels of this kind of dual-channel gastroscope are parallel, and it is not easy for the instruments placed within the two different channels to work together, therefore making it difficult to clip the nylon loop around the edge of the perforation [6]. Moreover, OTSC system, the Overstitch endoscopic suturing device and dual-channel endoscope are not usually available in most Chinese endoscopy units. Therefore, there are many limitations to their use and most endoscopists have less experience in such technologies.

In our case, after *en bloc* endoscopic full-thickness resection, we performed successful closure with a purse-string suture using the LeCamp™ endoloop and the endoclips fixed to the full thickness of the defect’s distal margin. Compared with the traditional nylon string, the LeCamp™ endoloop does not need to be preloaded and can be applied easily using the single channel endoscope. The endoclips fixed to the full thickness of the defect’s distal margin could prevent the clips from tearing the mucous membrane and shedding. This technique of endoscopic full-thickness resection combined with purse-string suture using the LeCamp™ endoloop through the common single-channel endoscope may be an alternative to avoid surgery for the removal of a giant upper esophagus tumor from muscularis propria layer.

## Abbreviations

ESE: Endoscopic submucosal excavation
OTSC: Over-the-scope clip
SMTs: Submucosal tumors
STER: Submucosal tunneling with endoscopic removal
VATS: Video assisted thoracoscopic surgery
